# Severe Iron-Deficiency Anemia due to Hookworm Hyperinfestation

**DOI:** 10.4269/ajtmh.25-0284

**Published:** 2025-08-26

**Authors:** Ravi Teja Reddy, Venkatesh Vaithiyam, Sanjeev Sachdeva

**Affiliations:** Department of Gastroenterology, GB Pant Hospital and Associated Maulana Azad Medical College, New Delhi, India

**Figure 1.**
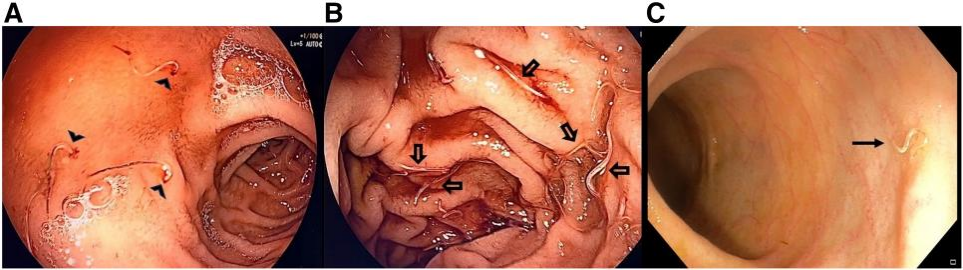
Endoscopic image shows multiple small slender worms with ingested blood adherent to the first part of the duodenum (arrowhead) (**A**), the second part of the duodenum (hollow arrow) (**B**) and the terminal ileum (solid arrow) (**C**).

A 20-year-old male residing in an Indian metropolitan city, with low socio-economic status and no prior comorbidities or addictions, presented with a 2-month history of progressive shortness of breath on exertion, palpitations, and fatigue. Physical examination showed tachycardia, pallor, and pedal edema, but no lymphadenopathy, jaundice, or organomegaly. Laboratory investigations revealed a hemoglobin level of 1.8 g/dL, total leukocyte count of 4.9 × 10^9^/L (differential leukocyte count: 50% neutrophils, 30% lymphocytes, 15% eosinophils, 5% monocytes), absolute eosinophil count of 735/*µ*L, and platelet count of 780,000/*µ*L. The patient’s total protein level was 6.9 g/dL, with albumin of 2.7 g/dL, and the rest of the liver function test and renal parameters were normal. Peripheral smear revealed microcytic hypochromic red cells and pencil cells suggestive of iron-deficiency anemia (IDA). His serum iron level (20 [normal range: 60–170 mcg/dL]) and ferritin level (10 [normal range 20–500 ng/mL]) were low, and his total iron-binding capacity (TIBC) (510 [normal range: 240–450 mcg/dL]) was increased, confirming IDA. Stool examination revealed ova of hookworms. Because of severe anemia and hypoalbuminemia, the patient underwent upper and lower gastrointestinal endoscopy to rule out other organic causes and was found to have multiple hookworms in the duodenum and terminal ileum (Figure 1). The patient was treated with intravenous iron, blood transfusions, and a 400 mg single dose of oral albendazole. At 6 weeks follow-up, the patient had significant clinical improvement, and his hemoglobin level increased to 8.5 g/dL

IDA is the most common form of anemia worldwide, especially in low-resource settings. The most common etiologies include chronic gastrointestinal blood loss, poor dietary intake, and parasitic infestations.^1^ The parasitic infections most associated with IDA are hookworm (Necator americanus and Ancylostoma duodenale), whipworm (Trichuris trichiura), malaria, and schistosomiasis. Hookworm infestation can cause chronic intestinal blood loss, intestinal inflammation, protein malabsorption, intestinal villous atrophy, and anorexia, leading to iron-deficiency anemia and hypoalbuminemia, as seen in our patient.^1^ Chronic exposure and reinfection in endemic areas can result in profound anemia requiring transfusions. Direct endoscopic visualization of hookworms is considered a useful diagnostic tool for unexplained anemia with suspected parasitic infections.^2^^,^^3^ Treatment involves stabilization with blood transfusion, iron therapy, and antihelminth drugs.
